# Integrative multi-omics analysis and machine learning refine global histone modification features in prostate cancer

**DOI:** 10.3389/fmolb.2025.1557843

**Published:** 2025-03-12

**Authors:** XiaoFeng He, QinTao Ge, WenYang Zhao, Chao Yu, HuiMing Bai, XiaoTong Wu, Jing Tao, WenHao Xu, Yunhua Qiu, Lei Chen, JianFeng Yang

**Affiliations:** ^1^ Department of Urology, Longhua Hospital, Shanghai University of Traditional Chinese Medicine, Shanghai, China; ^2^ Department of Urology, Fudan University Shanghai Cancer Center, Fudan University, Qingdao Institute of Life Sciences, Fudan University, Shanghai, China; ^3^ Shanghai Genitourinary Cancer Institute, Shanghai, China

**Keywords:** prostate cancer, histone modifications, epigenomics, multi-omics, machine learning, castration-resistant prostate cancer, immunotherapy

## Abstract

**Background:**

Prostate cancer (PCa) is a major cause of cancer-related mortality in men, characterized by significant heterogeneity in clinical behavior and treatment response. Histone modifications play key roles in tumor progression and treatment resistance, but their regulatory effects in PCa remain poorly understood.

**Methods:**

We utilized integrative multi-omics analysis and machine learning to explore histone modification-driven heterogeneity in PCa. The Comprehensive Machine Learning Histone Modification Score (CMLHMS) was developed to classify PCa into two distinct subtypes based on histone modification patterns. Single-cell RNA sequencing was performed, and drug sensitivity analysis identified potential therapeutic vulnerabilities.

**Results:**

High-CMLHMS tumors exhibited elevated histone modification activity, enriched proliferative and metabolic pathways, and were strongly associated with progression to castration-resistant prostate cancer (CRPC). Low-CMLHMS tumors showed stress-adaptive and immune-regulatory phenotypes. Single-cell RNA sequencing revealed distinct differentiation trajectories related to tumor aggressiveness and histone modification patterns. Drug sensitivity analysis showed that high-CMLHMS tumors were more responsive to growth factor and kinase inhibitors (e.g., PI3K, EGFR inhibitors), while low-CMLHMS tumors demonstrated greater sensitivity to cytoskeletal and DNA damage repair-targeting agents (e.g., Paclitaxel, Gemcitabine).

**Conclusion:**

The CMLHMS model effectively stratifies PCa into distinct subtypes with unique biological and clinical characteristics. This study provides new insights into histone modification-driven heterogeneity in PCa and suggests potential therapeutic targets, contributing to precision oncology strategies for advanced PCa.

## Introduction

Prostate cancer (PCa) is the second most frequently occurring cancer and remains one of the most commonly diagnosed malignancies in men, representing a significant cause of cancer-related mortality worldwide (Pujana-Vaquerizo et al., 2024). Current statistics reveal that 1 in every 44 men will succumb to prostate cancer, while 1 in 8 men will develop the disease at some point during their lifetime (Zeng et al., 2024). In 2024, projections estimate 299,010 new cases of prostate cancer, with approximately 35,250 deaths expected in the United States alone (INSTITUTE NC). The clinical presentation and course of PCa vary substantially among patients, spanning a spectrum from indolent, slow-growing tumors with minimal risk of progression, to highly aggressive variants defined by early recurrence and elevated cancer-specific mortality (Kulac et al., 2024). Most PCa are adenocarcinomas that originate from secretory glandular cells, and treatment guidelines recommend radical prostatectomy, radiotherapy, and hormone therapy as standard options for patients with localized or regional disease. These treatments have achieved remarkable success, with 5-year relative survival rates exceeding 99% for local and regional cases, as reported for men diagnosed between 2013 and 2019 (5). However, recurrence and progression to metastatic prostate cancer remain substantial challenges, significantly impacting patient outcomes and presenting ongoing difficulties for clinicians.

Recent advancements in histone modification research have profoundly influenced the field of cancer biology. Histone modifications are central to regulating chromatin structure and gene expression, thereby shaping cell fate and contributing to tumorigenesis. Key histone modifications, including methylation, acetylation, and phosphorylation, are pivotal regulators in the initiation and progression of various cancers. For example, SLC9A9 has been identified as a marker of abnormal histone acetylation in gastric cancer, functioning as an oncogenic factor (Guang et al., 2024). It modulates the positioning of the HBO1/KDM9 complex, influencing histone modification patterns of critical target genes such as LPCAT1, which promotes cholesterol synthesis and tumor progression (Yue et al., 2024). Similarly, histone-modifying enzymes such as PRMT6 and KDM5A have emerged as important players in diverse cancer types, underscoring their roles in tumor metabolism and immune evasion (Chen et al., 2024; He et al., 2024). Additionally, the co-expression of histone methyltransferases EZH2 and NSD2 has been associated with increased cancer aggressiveness and drug resistance, emphasizing the therapeutic potential of targeting histone modifications for early diagnosis and treatment (Xiong et al., 2024).

In recent years, the integration of multi omics data—including genomics, transcriptomics, epigenomics, and proteomics—with machine learning (ML) technologies has significantly advanced cancer research (Menyhárt and Győrffy, 2021). multi omics provides a comprehensive view of the molecular basis of cancer, elucidating the complex interactions among genetic, epigenetic, and proteomic levels that drive tumorigenesis (Valous et al., 2024). Machine learning methods, particularly deep learning and ensemble algorithms, excel at handling high-dimensional datasets, detecting hidden patterns, and offering predictive insights for clinical decision-making (Cai et al., 2022; Vahabi and Michailidis, 2022). Moreover, unsupervised learning techniques such as autoencoders and variational autoencoders are employed to extract potential representations from multi omics datasets, facilitating the discovery of underlying patterns by learning compressed representations of the data. Supervised learning methods can be trained using input data and predefined output tags to identify features associated with disease phenotypes (Simidjievski et al., 2019). These advancements empower data-driven biomedical research to elucidate the molecular mechanisms of cancer with greater resolution and accuracy. Notably, the challenge of effectively integrating multiple modalities to address issues of incompleteness, sparsity, and high dimensionality has emerged as a key concern in multi omics analysis.

In PCa, histone modifications have garnered growing attention for their critical role in disease progression. Histone demethylases and methyltransferases, including KDM5A, KDM5B, and EZH2 (Du et al., 2020), are particularly implicated in advanced prostate cancer and castration-resistant prostate cancer (CRPC). For instance, KDM5A facilitates PCa progression by hyperactivating the PI3K/AKT signaling pathway, while KDM5B influences tumor metabolism and cell proliferation via epigenetic regulation (Rodems et al., 2022; Li et al., 2020). In CRPC, NSD2 upregulation induces epigenetic alterations, such as gains in H3K36me2 and losses in H3K27me3, coupled with shifts in chromatin compartmentalization from inactive to active states, collectively contributing to prostate carcinogenesis (Kanaoka et al., 2024). Similarly, MAT2A enhances H3K4me2 at multiple loci, driving the expression of pro-tumorigenic, non-canonical androgen receptor (AR) target genes (Cacciatore et al., 2024). These findings underline the pivotal role of histone modifications and chromatin remodeling in shaping the molecular landscape of PCa.

Despite significant progress in elucidating the roles of histone modifications in cancer, many studies remain focused on isolated modification patterns or specific genes, leaving a critical gap in the comprehensive understanding of global histone modification networks. Current research often emphasizes individual histone modification types, such as methylation, acetylation, phosphorylation, or ubiquitination, while neglecting their interactions and synergistic effects across diverse genomic regions. This reductionist approach limits our ability to fully comprehend the global regulatory networks of histone modifications and their collective impact on complex diseases like cancer. An integrative exploration of global histone modification patterns is thus essential to unravel the intricate epigenetic mechanisms underlying prostate cancer progression and to identify novel therapeutic targets.

## Methods and materials

### Data collection, preprocessing, and patient summary

The gene expression matrix and comprehensive clinical data of PCa patients were obtained from three independent publicly available databases: The Cancer Genome Atlas (TCGA, https://portal.gdc.cancer.gov/), the Gene Expression Omnibus (GEO, https://www.ncbi.nlm.nih.gov/geo/), and ArrayExpress (https://www.ebi.ac.uk/arrayexpress/). A total of 838 samples were ultimately included from three cohorts, specifically TCGA-PRAD (n = 495), MSKCC (n = 140), and GSE70770 (n = 203). Gene sequencing results from the three cohorts were expressed in transcripts per million (TPM) formats, and the expression data were pre-transformed to log2 (TPM +1) for comparability. Noise was defined as mRNAs with a TPM value <1 in over 90% of the samples, which were subsequently removed. Patients without paired mRNA profiles or clinical information, as well as those lacking follow-up time, were excluded to mitigate potential bias. Recurrence-free survival (RFS) was designated as the outcome variable. After pretreatment, gene expression data from three cohorts were merged by aligning the common genomes present in all datasets. This was achieved by intersecting the genomes of the three cohorts to ensure consistency in the analysis. To minimize batch effects and potential technical differences among the three cohorts, we employed the ComBat method from the R package ‘sva’ for batch effect correction (Anwaier et al., 2023). This step ensures that any systematic differences arising from the source of the datasets are corrected, resulting in more accurate downstream analyses. Consequently, a total of 838 samples were identified for further analysis, and the detailed clinicopathological features of these patients are presented in Table 1.

**TABLE 1:** The distribution of clinicopathological features among TCGA-PRAD, GSE70770, and MSKCC cohorts.

	GSE70770 (N=203)	MSKCC (N=140)	TCGA-PRAD (N=495)	Overall (N=838)
Survival time				
Mean (SD)	40.2 (27.6)	46.0 (30.3)	31.5 (24.8)	36.1 (27.1)
Median [Min, Max]	36.7 [0.362, 103]	45.5 [1.38, 149]	25.8 [0.750, 165]	30.4 [0.362, 165]
Age				
Mean (SD)	NA (NA)	58.1 (6.97)	61.0 (6.84)	60.4 (6.96)
Median [Min, Max]	NA [NA, NA]	58.0 [37.3, 83.0]	61.0 [41.0, 78.0]	60.9 [37.3, 83.0]
Missing	203 (100%)	0 (0%)	0 (0%)	203 (24.2%)
Stage				
T2	79 (38.9%)	86 (61.4%)	187 (37.8%)	352 (42.0%)
T3	118 (58.1%)	47 (33.6%)	291 (58.8%)	456 (54.4%)
T4	1 (0.5%)	7 (5.0%)	10 (2.0%)	18 (2.1%)
Unknown	5 (2.5%)	0 (0%)	7 (0%)	14 (0.6%)
Gleason				
10	1 (0.5%)	0 (0%)	4 (0.8%)	5 (0.6%)
5	2 (1.0%)	0 (0%)	0 (0%)	2 (0.2%)
6	35 (17.2%)	41 (29.3%)	45 (9.1%)	121 (14.4%)
7	140 (69.0%)	76 (54.3%)	246 (49.7%)	462 (55.1%)
8	13 (6.4%)	11 (7.9%)	63 (12.7%)	87 (10.4%)
9	10 (4.9%)	10 (7.1%)	137 (27.7%)	157 (18.7%)
Unknown	2 (1.0%)	2 (1.4%)	0 (0%)	4 (0.5%)

### Estimation of global histone modification patterns among PCa patients

In order to clarify the features of histone modifications in PCa, we gathered 122 signaling pathways related to histone modifications from the Molecular Signatures Database (MSigDB), particularly those listed under the C5 ontology gene sets (Liberzon et al., 2011). The biological processes we examined included histone-mediated phosphorylation, methylation, ubiquitination, and acetylation. We utilized the software “GSVA, v.3.5″to evaluate the activation status of these 122 pathways. For every sample within the TCGA-PRAD cohort, we computed the enrichment score for each individual gene set to measure the overall activation extent of that gene set (Hänzelmann et al., 2013). As a result, the transcript profiles obtained from the TCGA-PRAD cohort were analyzed through the lens of gene set activation profiles.

### Identification of differentially expressed genes (DEGs) among different histone modification patterns

By applying a predetermined threshold of p < 0.05 and |log2fc| > 1, we identified differentially expressed genes (DEGs) between the two clusters with the help of the “limma” package. Additionally, the “clusterProfiler 4.0” R package was employed to explore the downstream signaling pathways linked with the DEGs, annotated according to Gene Ontology (GO) and Kyoto Encyclopedia of Genes and Genomes (KEGG) (Wu et al., 2021). The fast gene set enrichment analysis (fgsea) algorithm implemented in R package “fgsea” was performed for HALLMARKE annotation.

### Construction of consensus machine learning based histone modification risk model

To ensure the robustness of the machine learning-based consensus model, a variety of strategies were adopted. First, prognostic genes were identified through univariate COX regression analysis in three independent cohorts: TCGA-PRAD, GSE70770, and MSKCC. These genes were categorized into high and low expression groups based on median expression values and subjected to further analysis. Only genes with P values <0.05 and hazard ratios (HR) > 1 were deemed significant risk or protective factors. Secondly, to enhance the reliability of the identified prognostic genes, ten different machine learning algorithms were employed, including Random Survival Forest (RSF), Elastic Net (ENET), Lasso, Ridge, Stepwise COX Regression, Coxboost, Partial Least Squares Regression Cox (PLSRCOX), Supervised Principal Component (SUPERPC), Generalized Boosted Modeling (GBM), and Survival Support Vector Machine (Survival-SVM). These algorithms were selected for their complementary advantages in handling high-dimensional, multivariate data, and were applied to create integrated, robust consensus models. To further ensure the model’s universality, the leave-one-out cross-validation (LOOCV) method was utilized to evaluate the performance of 101 different combinations of algorithms. LOOCV minimizes overfitting by training on multiple subsets of the data while testing on the remaining data, thereby ensuring that the model is consistently executed across various training and validation schemes. The TCGA-PRAD dataset serves as the training set (n = 495), whereas the MSKCC (n = 140) and GSE70770 (n = 203) datasets are utilized as the external validation sets (Jiang et al., 2024) (Liu et al., 2022). The consistency index (C-index) for each model is calculated across all external validation datasets to assess its predictive accuracy. A higher C-index indicates greater reliability and robustness of the model in differentiating between high-risk and low-risk patients. Through this meticulous process, a consensus machine is developed to learn histone modification signatures (CMLHM), thereby enhancing the model’s reliability in predicting patient outcomes, and the risk score was calculated as following formula:

$$CMLHM\;score=\sum_{k=1}^{n}\left(coef.i*expression.i\right)$$

### Multivariate Cox regression analysis

All of the patients were separated into high and low score subgroups with the median CMLHM score. K-M plot and was used to assess the prognostic value of CMLHMS, and receiver operating characteristic (ROC) curve was employed to evaluate the discrimination ability of the model. After adjusting the intrinsic impact of variates via the Cox proportional hazard-regression model, multivariate analysis was performed for the prognostic value of CMLHM and clinicopathological features.

### Cell lines and cell culture

The prostate cancer cell lines C4-2 and 22RV1 were obtained from the American Type Culture Collection (ATCC) and cultured in RPMI-1640 medium supplemented with 10% fetal bovine serum (FBS) and 1% penicillin-streptomycin at 37°C in a humidified incubator containing 5% CO_2_. All cell lines were authenticated using short tandem repeat (STR) profiling and routinely tested for *mycoplasma* contamination.

### siRNA transfection

Small interfering RNAs (siRNAs) targeting PRC1 (siRNA1 and siRNA2) and a non-targeting negative control siRNA (NC) were purchased from GenePharma (Shanghai, China). Transfections were performed using Lipofectamine RNAiMAX (Invitrogen, United States) according to the manufacturer’s protocol. Briefly, cells were seeded in six-well plates and transfected with 50 nM siRNA for 48 h. Knockdown efficiency was assessed by Western blot.

### Western blot analysis

Cells were lysed using RIPA buffer supplemented with protease inhibitors (Roche, Switzerland). Protein concentration was determined using the BCA Protein Assay Kit (Thermo Fisher Scientific, United States). Equal amounts of protein were separated by SDS-PAGE and transferred to PVDF membranes (Millipore, United States). Membranes were blocked with 5% non-fat milk in TBST and incubated overnight at 4°C with primary antibodies against PRC1 (Abcam, ab181147) and GAPDH (CST, 5,174). After washing, membranes were probed with horseradish peroxidase (HRP)-conjugated secondary antibodies and visualized using ECL detection reagents (Thermo Fisher Scientific). Band intensities were quantified using ImageJ software, and PRC1 levels were normalized to GAPDH.

### Cell proliferation assay

Cell proliferation was evaluated using the Cell Counting Kit-8 (CCK-8, Dojindo, Japan). Transfected C4-2 and 22RV1 cells were seeded in 96-well plates at a density of 3 × 10^3^ cells per well. CCK-8 reagent was added at 24-h intervals over 5 days, and optical density (OD) was measured at 450 nm using a microplate reader (BioTek, United States). Experiments were performed in triplicate, and data were normalized to the control group.

### Transwell migration assay

Cell migration was assessed using transwell chambers (8-μm pore size, Corning, United States). At 48 h post-transfection, 2 × 10^4^ cells in serum-free medium were seeded into the upper chamber, while the lower chamber contained medium supplemented with 10% FBS as a chemoattractant. After 24 h of incubation at 37°C, non-migrating cells were removed from the upper side of the membrane, and migrated cells on the lower surface were fixed with 4% paraformaldehyde and stained with 0.1% crystal violet. Images were captured using a light microscope (Nikon, Japan), and migrated cells were counted in five random fields per well. Data represent the average of three independent experiments.

### Chemotherapeutic response evaluation

Regarding the initial chemotherapy regimen for prostate cancer (PCa) and the recognized activation of signaling pathways, we chose particular medications to assess the predictive therapeutic capabilities of our model. The pertinent data was obtained from GDSC 2016 (https://www.cancerrxgene.org/) and then incorporated into the ComDrug program found in the “MOVICS” package (Lu et al., 2020). For each patient, we utilized ridge regression analysis to calculate the estimated inhibitory concentration (IC50), reflecting their responses to different drugs.

### Single cell RNA sequencing analysis

Transcriptome data for single cells were derived from the PRJNA699369 cohort (Guang et al., 2024). This study reveals that a small population of cells in primary prostate cancer exhibits characteristics of castration-resistant prostate cancer (CRPC) even before the initiation of hormone therapy, indicating that these cells are inherently castration-resistant. In this investigation, we primarily utilized epithelial cells from seven samples, which included three primary prostate cancer samples and four CRPC samples. Data preprocessing of raw information was carried out utilizing the Seurat package (Stuart et al.). This process included the removal of cells that had fewer than 200 or more than 2,500 transcripts detected, along with those presenting mitochondrial gene percentages greater than 10%. To address cell cycle influences on single-cell transcriptomic data, the CellCycleScoring function within Seurat was used for scoring cell cycles. For normalization and logarithmic transformation, the NormalizeData function, which employs the LogNormalize method, was applied. The identification of highly variable genes was accomplished with the FindVariableFeatures function (avg_log2FC > 0.3 and adjusted P < 0.05), utilizing the variance stabilizing transformation (vst) approach, preserving the top 2,000 genes with the greatest variability. Following this, batch effects across samples were addressed using the ScaleData function. After preprocessing the data, dimensionality reduction was performed to enable further analysis of high-dimensional single-cell transcriptomic data. The RunPCA function in Seurat facilitated principal component analysis (PCA), the most commonly used approach, while retaining the leading 50 principal components. Subsequently, inter-cell K-nearest neighbor (KNN) relationships were identified using the FindNeighbors function, and cell clustering was executed through the Louvain method via the FindClusters function. The annotation of the cell clusters was conducted manually, based on recognized signature markers (Cheng et al., 2022; Song et al., 2022; Messex and Liou, 2023).

### Slingshot analysis

To create cellular trajectories, we used the Slingshot algorithm for cell lineage inference (Street et al., 2018). This algorithm organizes cells along their developmental paths to forecast lineage trajectories and bifurcations. We applied the default configurations from the Slingshot package in R, using Seurat’s UMAP coordinates and cluster assignments as our input data.

### Immunohistochemistry (IHC) staining analysis

Tissue from two patients (Xu et al., 2020) who had undergone either radical or partial nephrectomy at the Department of Urology, Longhua Hospital, Shanghai University of Traditional Chinese Medicine, were chosen for this analysis, including patients diagnonsed as low grade PCa (Gleason score: 3 + 4 = 7, T2N0M0) and high grade PCa (Gleason score: 4 + 4 = 8, T3N1M0). We conducted IHC staining to evaluate the expression of PRC1. Samples were blocked with blocking buffer (1.5 h, 22°C), incubated with PRC1 polyclonal antibody (Product # PA5-101025, ThermoFisher, United States) using a dilution of 1:100 (1.5 h, 22°C), followed by HRP conjugated goat anti-rabbit. Detailed IHC procedures could refer to our prior studies (Yin et al., 2019; Ma et al., 2024). Tumor specimens were acquired and maintained in a 4% formaldehyde solution for a duration of 24 h. After this period, the specimens were embedded in paraffin and cut into slices approximately 5 μm thick. The sections of the tumor underwent deparaffinization and rehydration, which was followed by the inhibition of endogenous peroxidase activity and the retrieval of antigens. Subsequently, a 5% BSA solution was introduced to the tumor sections to reduce non-specific binding for 30 min; they were then incubated with primary antibodies overnight. After a 1-h incubation period with secondary antibodies, visualization of the tumor sections was performed using a DAB kit.

### Statistical analysis

The survival outcomes were compared by the log-rank test, the categorical data were analyzed via Fisher’s exact test. The distribution between the high-CMLHM and low- CMLHM subgroups were compared by Student’s t-test. All statistical analyses were performed using R (Version: 4.2.1). A two-tailed *p*-value <0.05 was recognized as statistically significant.

## Results

### Distinctive global histone modification patterns in prostate cancer

**FIGURE 1:**
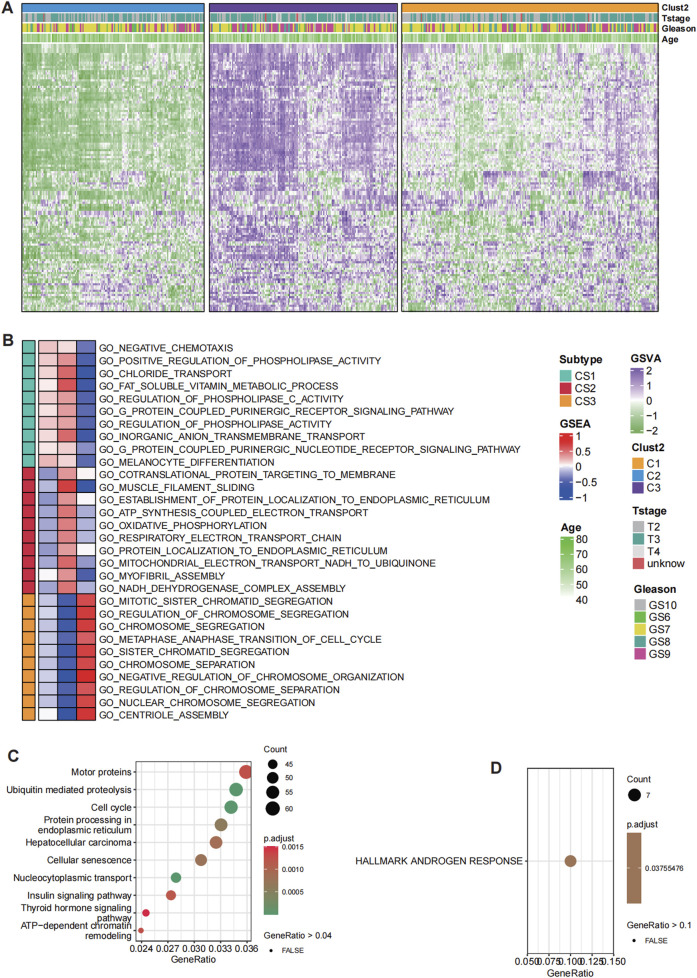
Distinct histone modification patterns in prostate cancer (PCa). **(A)** Hierarchical clustering of patients in the TCGA-PRAD cohort based on histone modification GSVA scores identified three clusters with distinct histone modification patterns. **(B)** GO enrichment analysis for C1, C2 and C3. **(C)** KEGG pathway analysis for deferentially expressed genes (DEGs) between C3 and C2. **(D)** HALLMARKS pathway analysis based on 50 classical oncological pathways for DEGs between C3 and C2.

By leveraging 122 signaling pathways associated with histone modifications, we identified distinct global histone modification patterns in prostate cancer (PCa) that highlight their potential role in driving tumor heterogeneity. Using the TCGA-PRAD cohort, we calculated histone modification scores and performed hierarchical clustering via the distance matrix function from the “ClassDiscovery” package. This analysis stratified patients into three discrete clusters, each characterized by unique histone modification profiles (Figure 1A). Among these, cluster C3 exhibited the highest degree of histone modification activation, while cluster C2 demonstrated a markedly subdued state, reflecting diverse epigenetic landscapes across the clusters.

Notably, cluster C3 displayed the highest level of histone modification activation, whereas cluster C2 exhibited a significantly subdued state, revealing diverse regulatory environments among the clusters. The biological implications of these clusters were further explored via pathway analysis, which identified significant variations in functional activation. Pathways linked to mitotic sister chromatid separation, chromosome segregation, and the regulation of chromosome segregation were particularly enriched in cluster C3, indicating a vigorous proliferative phenotype associated with increased genomic instability. This suggests that the cells within cluster C3 are in a state of active division, where chromosomal dynamics and regulation during mitosis are crucial to their biological function. Conversely, cluster C2 was marked by heightened metabolic activity, incorporating pathways that play roles in cellular metabolism, protein production, energy generation, and structural maintenance. This indicates a relatively quiescent phase concentrated on maintaining cellular balance and metabolic processes (Figure 1B).

Interestingly, cluster C3 also showed heightened activation in pathways associated with chemotaxis, phospholipid metabolism, ion transport, vitamin metabolism, cell signaling, and differentiation. These pathways suggest a broader involvement in cellular communication, migration, and specialization, which may contribute to tumor progression and interactions within the tumor microenvironment. In comparison, the subdued nature of these pathways in cluster C2 further supports its metabolic and maintenance-oriented phenotype. To refine the essential differences between clusters C3 and C2, KEGG pathway analysis was conducted (Figure 1C). Among the most significantly enriched pathways, androgen response was identified as the key differentiator between these two clusters. The robust androgen response observed in cluster C3 suggests dependency on androgen receptor (AR)-mediated transcriptional programs, which are critical for PCa progression. Conversely, the diminished androgen response in cluster C2 implies a metabolic adaptation divergent from AR-driven phenotypes, potentially reflecting an alternative survival strategy.

Our findings reveal distinct histone modification clusters in PCa, each representing unique regulatory and functional states. Cluster C3 is characterized by proliferative and androgen-driven dynamics, while cluster C2 exhibits a quiescent, metabolically focused phenotype. These contrasting profiles underscore the role of histone modifications in shaping tumor biology and highlight potential therapeutic opportunities for targeted interventions. The interplay between genomic instability, metabolic adaptations, and androgen signaling offers critical insights into prostate cancer progression and treatment resistance (Figure 1D). Overall, the identification of distinct histone modification clusters in PCa underscores the profound epigenetic heterogeneity within tumors, revealing divergent proliferative and metabolic phenotypes. These findings not only highlight the critical role of histone modifications in shaping tumor biology but also provide a foundation for developing precision therapeutic strategies targeting specific epigenetic and androgen-driven vulnerabilities.

### High CMLHMS score indicates poor prognosis in prostate cancer

**FIGURE 2:**
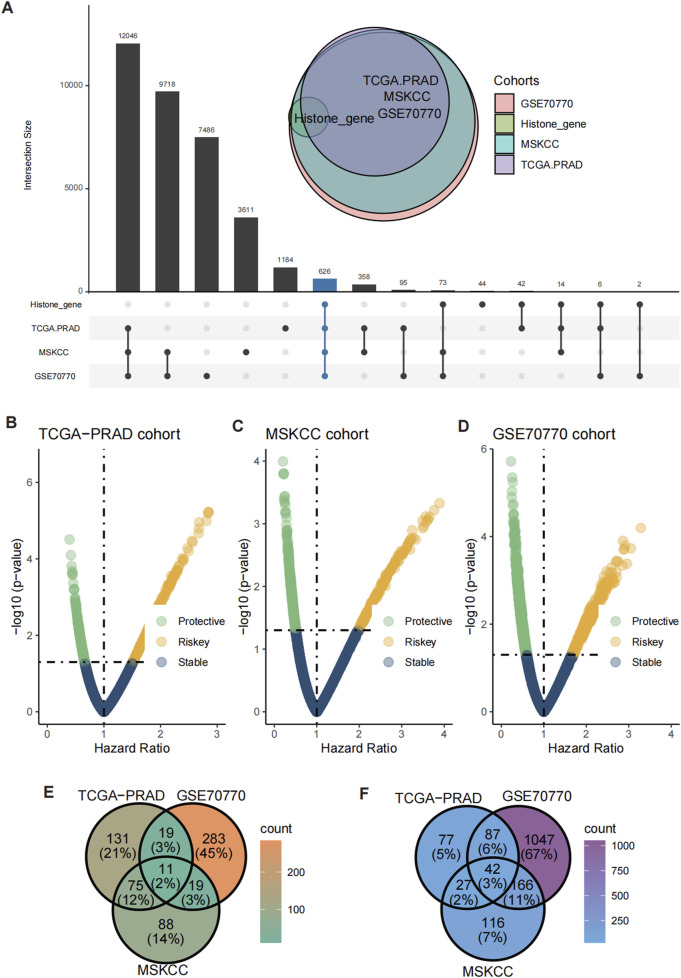
Identification of shared risky and protective genes. **(A)** UpSet plot showed the overlap of histone modification-related genes and DEGs across the TCGA-PRAD, GSE70770, and MSKCC cohorts. **(B–D)** Volcano plots showing the distribution of risky and protective genes in the TCGA-PRAD **(B)**, GSE70770 **(C)**, and MSKCC **(D)** cohorts. **(E, F)** Heatmaps illustrating the expression patterns of 11 risky genes **(E)** and 42 protective genes **(F)** shared across the three cohorts.

**FIGURE 3:**
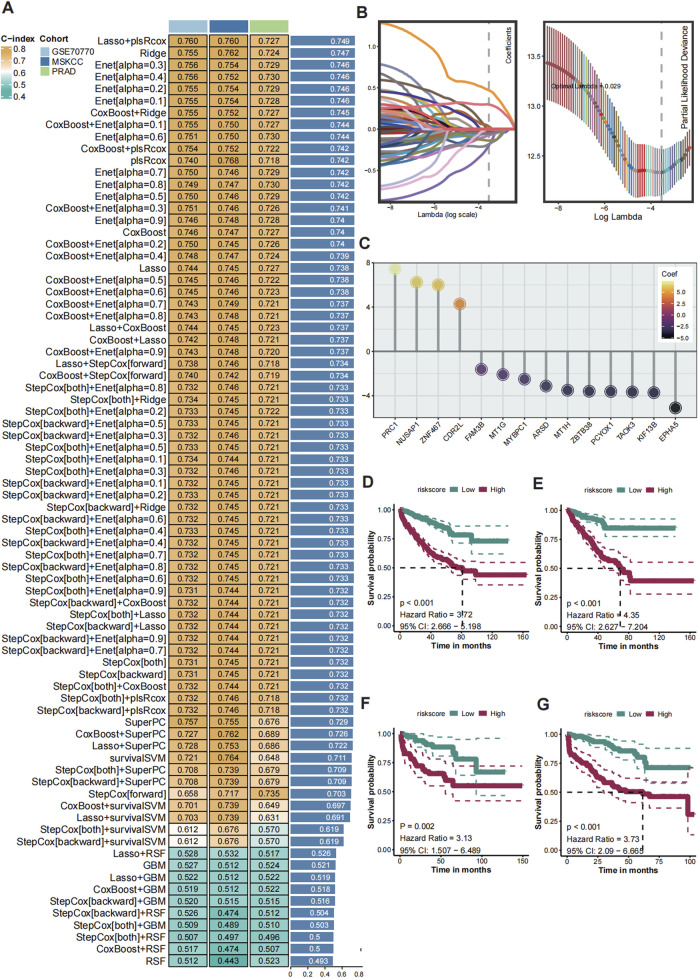
Construction of the consensus machine learning histone modification signature. **(A)** The heatmap showed the 101 distinct prediction models based on LOOV framework, and their C-index were calculated comprehensively across the TCGA-PRAD, GSE70770, and MSKCC cohorts. **(B)** The coefficient profiles of the candidate genes are plotted against the log-transformed lambda values and the partial likelihood deviance. **(C)** Univariate Cox regression analysis showing HRs for the 14 model genes, with PRC1 identified as the top risk factor. **(D–G)** Kaplan-Meier (K–M) survival curves of CMLHMS scores in the merged cohort **(D)**, TCGA-PRAD **(E)**, GSE70770 **(F)**, and MSKCC **(G)**, demonstrating that high CMLHMS scores are significantly associated with shorter RFS.

The UpSet plot revealed a significant overlap of histone modification-related genes across the TCGA-PRAD, MSKCC, and GSE70770 cohorts, with 626 shared differentially expressed genes (DEGs) identified (Figure 2A). To further assess the prognostic significance of these genes, univariate Cox regression analysis was conducted, categorizing genes into risky and protective groups across the three datasets (Figures 2B–D). Ultimately, 11 risky genes (Figure 2E) and 42 protective genes (Figure 2F) were consistently identified among the cohorts and subsequently selected for input into the leave-one-out validation (LOOV) machine learning process (Figure 3A).

To construct a robust consensus machine learning model based on global histone modification features, batch effects among the TCGA-PRAD, MSKCC, and GSE70770 cohorts were corrected (Supplementary Figure S1). The TCGA-PRAD dataset was employed as the training cohort, while the MSKCC and GSE70770 datasets served as validation cohorts. Using the leave-one-out cross-validation (LOOCV) framework, 101 machine learning prediction models were developed, and the concordance index (C-index) was calculated for each model across all validation datasets. Notably, the optimal model was identified as a combination of LASSO and plsRcox, achieving the highest average C-index of 0.749.

From this model, 14 genes were established as part of the final signature, including four risky genes and ten protective genes (Figure 3B). These genes were further analyzed using univariate Cox regression, which revealed that PRC1, CDR2L, NUSAP1, and ZNF467 function as risky factors in PCa, with elevated PRC1 levels presenting the highest risk (Figure 3C). Based on these findings, the Comprehensive Machine Learning Histone Modification Score (CMLHMS) was developed using the formula: CMLHMS Score = (0.057514 * PRC1) + (0.232672 * CDR2L) + (0.333656 * NUSAP1) + (0.217279 * ZNF467) − (0.177885 * ARSD) − (0.096998 * PCYOX1) + (0.028384 * TAOK3) − (0.167867 * ZBTB38) − (0.140445 * KIF13B) − (0.106067 * EPHA5) − (0.104789 * FAM3B) − (0.058782 ∗ MT1G) − (0.032181 * MYBPC1) − (0.003047 * MT1H).

The newly developed signature demonstrated strong prognostic value for predicting prostate cancer recurrence. In the merged cohort, patients with high CMLHMS scores exhibited significantly worse recurrence-free survival (RFS) (P < 0.001, HR = 3.72, 95% CI: 2.666–5.198). Further validation in individual cohorts confirmed these results, with high CMLHMS scores associated with shorter RFS in TCGA-PRAD (P < 0.001, HR = 4.35, 95% CI: 2.627–7.204), MSKCC (P = 0.002, HR = 3.13, 95% CI: 1.507–6.489), and GSE70770 (P < 0.001, HR = 3.73, 95% CI: 2.09–6.665) (Figures 3D–G). Collectively, these findings indicate that a high CMLHMS score correlates with a 3- to 4-fold increase in the likelihood of shorter RFS, underscoring its utility as a prognostic biomarker. This robust model offers a novel avenue for predicting disease progression and stratifying PCa patients for tailored therapeutic interventions.

### High PRC1 expression correlates with high malignancy and poor prognosis

**FIGURE 4:**
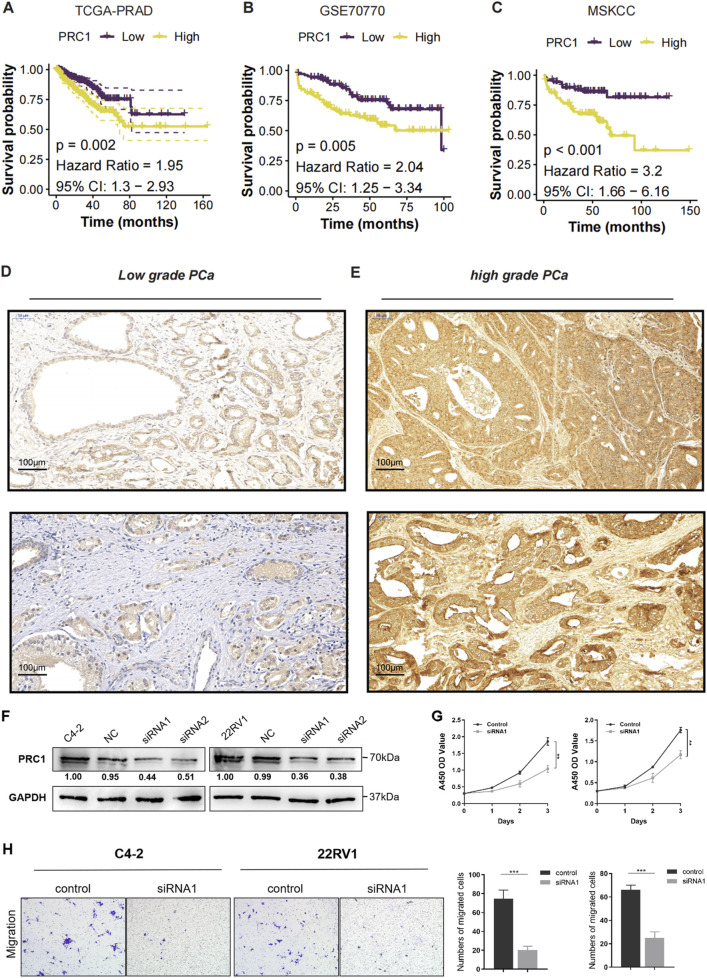
High PRC1 Expression Correlates with High Malignancy and Poor Prognosis. **(A–C)** K-M survival curves for PRC1 expression in the TCGA-PRAD **(A)**, GSE70770 **(B)**, and MSKCC **(C)** cohorts. **(D)** Immunohistochemical (IHC) staining images of PRC1 in low-grade PCa. **(E)** Immunohistochemical (IHC) staining images of PRC1 in high-grade PCa. **(F)** Western blot analysis confirmed efficient knockdown of PRC1 expression using two independent siRNAs (siRNA1 and siRNA2) in C4-2 and 22RV1 cell lines, compared to negative control (NC). **(G)** Cell proliferation was assessed using the CCK-8 assay over 5 days. PRC1 knockdown significantly inhibited the proliferation of both C4-2 and 22RV1 cells compared to controls. **(H)** Transwell migration assays showed a marked decrease in the migratory ability of PRC1 knockdown cells compared to controls. Quantification of migrated cells revealed significant reductions in both C4-2 and 22RV1 cell lines upon PRC1 silencing.

Given that PRC1 exhibited the highest linear and Cox regression coefficients among the four signature genes, it was deemed the most critical contributor to the CMLHMS model and subjected to further in-depth analysis. To evaluate its prognostic value, Kaplan-Meier survival curves were generated across the three cohorts. The results consistently demonstrated that elevated PRC1 expression was significantly associated with poorer survival outcomes in all datasets. In the TCGA-PRAD cohort (Figure 4A), patients with high PRC1 expression had a hazard ratio (HR) of 1.95 (95% CI: 1.3–2.93, P = 0.002), indicating significantly shorter recurrence-free survival (RFS). Similarly, in the GSE70770 cohort (Figure 4B), high PRC1 expression was linked to worse survival outcomes, yielding an HR of 2.04 (95% CI: 1.25–3.34, P = 0.005). In the MSKCC cohort, the association was even more pronounced, with an HR of 3.2 (95% CI: 1.66–6.16, P < 0.001) (Figure 4C).

To further validate the association between PRC1 expression and tumor severity, IHC staining was performed on low-grade and high-grade prostate cancer tissues (Figures 4D, E). The results revealed that low-grade PCa tissues exhibited weak and sparse PRC1 staining, whereas high-grade PCa tissues displayed significantly higher PRC1 expression, characterized by widespread and intense staining. These findings suggest a strong correlation between PRC1 and tumor aggressiveness, as reflected by Gleason scores. Furthermore, functional assays were conducted to evaluate PRC1’s role in prostate cancer progression. Western blot analysis confirmed efficient knockdown of PRC1 expression using two siRNAs (siRNA1 and siRNA2) in the C4-2 and 22RV1 cell lines (Figure 4F). CCK-8 assays revealed that PRC1 knockdown significantly suppressed cell proliferation in both cell lines (Figure 4G). Similarly, transwell migration assays demonstrated a marked reduction in migratory ability upon PRC1 knockdown, with significantly fewer migrated cells observed in PRC1-silenced groups compared to controls (Figure 4H, P < 0.001).

In addition, we performed K-M survival analyses for the other three signature genes (CDR2L, NUSAP1, and ZNF467) across the three cohorts. Consistent results were observed, showing that elevated expression of CDR2L, NUSAP1, and ZNF467 was also significantly associated with poor clinical outcomes (Supplementary Figures S2A–C, all P < 0.05). Collectively, these findings highlight PRC1 as a critical biomarker of poor prognosis in prostate cancer, with strong associations to shorter RFS and higher Gleason scores. Its consistent correlation with aggressive disease suggests PRC1’s potential as a therapeutic target, particularly in advanced or high-grade PCa.

### CMLHMS serves as an independent risk factor in prostate cancer

**FIGURE 5:**
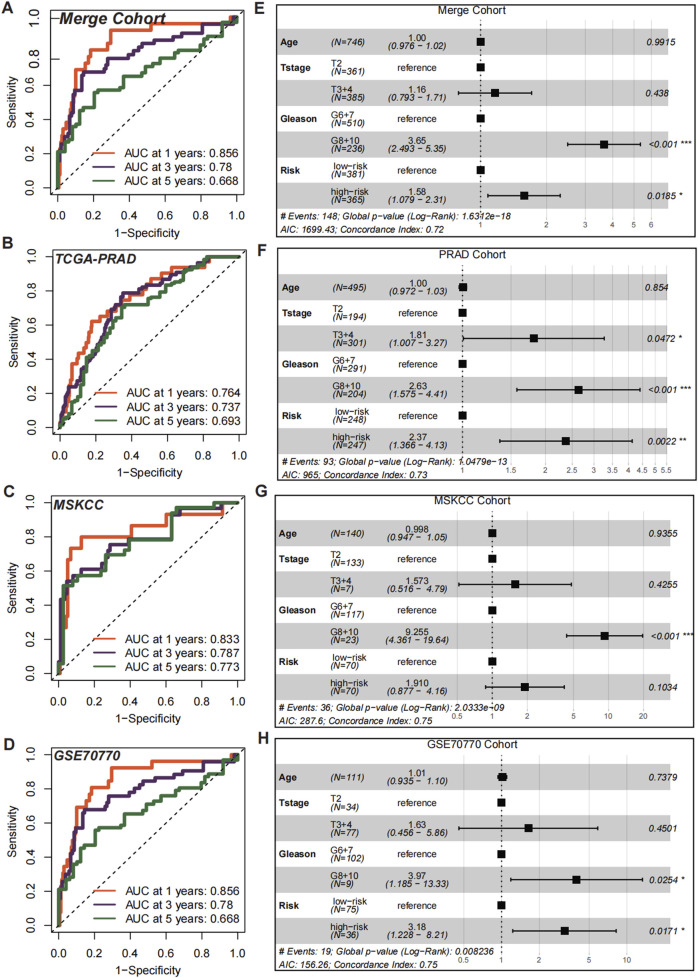
Prognostic and independent risk factor evaluation of CMLHMS. **(A–D)** Receiver operating characteristic (ROC) curves for the CMLHMS model in predicting 1-, 3-, and 5-year RFS in the merged cohort **(A)**, TCGA-PRAD **(B)**, MSKCC **(C)**, and GSE70770 **(D)**. The model demonstrates strong discriminatory power across all cohorts. **(E–H)** Multivariate Cox regression analyses for CMLHMS scores and clinicopathological feature **(E)**, TCGA-PRAD **(F)**, MSKCC **(G)**, and GSE70770 **(H)**.

To evaluate the discriminatory efficiency of CMLHMS, ROC analysis was conducted across the merged cohort, TCGA-PRAD, MSKCC, and GSE70770 cohorts, revealing favorable performance with 1-, 3-, and 5-year AUCs of 0.856, 0.780, and 0.668 in the merged cohort (Figure 5A); 0.764, 0.737, and 0.693 in the TCGA-PRAD cohort (Figure 5B); 0.833, 0.787, and 0.773 in the MSKCC cohort (Figure 5C); and 0.856, 0.780, and 0.668 in the GSE70770 cohort (Figure 5D), respectively. These results highlight the consistent prognostic accuracy of CMLHMS across multiple datasets, particularly in short-to medium-term predictions.

To further establish the independent prognostic significance of CMLHMS, multivariate Cox regression analyses were conducted. In the merged cohort (Figure 5E), both high Gleason scores (8, 9, and 10; HR = 3.65, 95% CI: 2.493–5.35, P < 0.001) and high CMLHMS scores (HR = 1.58, 95% CI: 1.079–2.31, P = 0.0185) were identified as significant risk factors for shorter RFS. In the TCGA-PRAD cohort (Figure 5F), T stages 3 and 4 (HR = 1.81, 95% CI: 1.007–3.27, P = 0.0472), high Gleason scores (HR = 2.63, 95% CI: 1.575–4.41, P < 0.001), and high CMLHMS scores (HR = 2.37, 95% CI: 1.366–4.13, P = 0.0022) were all significant predictors. In the MSKCC cohort (Figure 5G), high Gleason scores (HR = 9.255, 95% CI: 4.361–19.54, *P* < 0.001) were statistically significant, while CMLHMS scores (HR = 1.91, 95% CI: 0.877–4.16, *P* = 0.1034) indicated a clear trend toward increased risk. In the GSE70770 cohort (Figure 5H), both high Gleason scores (HR = 3.97, 95% CI: 1.185–13.33, *P* = 0.0254) and high CMLHMS scores (HR = 3.18, 95% CI: 1.22–8.21, *P* = 0.0171) were identified as independent risk factors. These findings underscore the robust discriminatory efficiency of CMLHMS and its role as a reliable independent risk factor in PCa, highlighting its significant prognostic value and potential utility in guiding risk stratification.

### Divergent biological pathways define high- and Low-CMLHMS PCa subtypes

**FIGURE 6:**
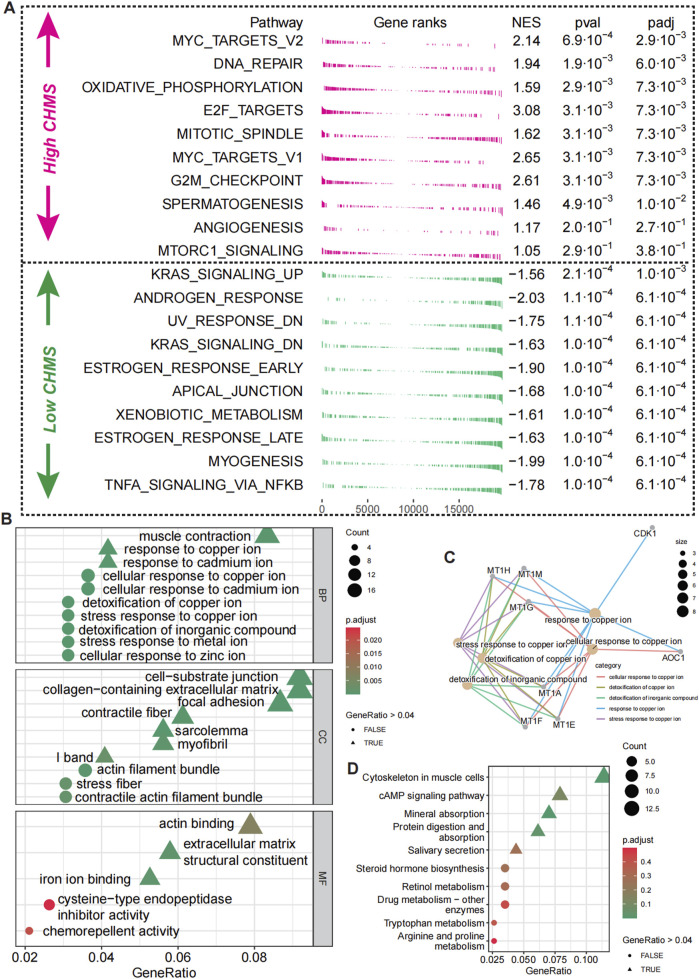
Biological pathway differences in high- and low-CMLHMS PCa. **(A)** Fgsea analysis of hallmark pathways for high-CMLHMS an low-CMLHMS score tumors. **(B, C)** GO enrichment analysis. **(D)** KEGG analysis.

To delineate the distinct molecular landscapes between prostate cancer (PCa) patients with high and low CMLHMS, we conducted an enrichment analysis of DEGs (Supplementary Figure S2D). The forest GESA analysis, utilizing 50 hallmark signaling pathways (Figure 6A), revealed profound differences in pathway activation between the two groups, underscoring unique tumor biology. High-CMLHMS tumors demonstrated pronounced activation of pathways known to drive invasiveness and proliferation, including MYC TARGETS V2, OXIDATIVE PHOSPHORYLATION, E2F TARGETS, G2M CHECKPOINT, and ANGIOGENESIS. These findings suggest a molecular signature geared toward rapid cell cycle progression, energy metabolism, and vascular remodeling, consistent with a highly aggressive tumor phenotype.

To further refine these observations, functional enrichment analysis using GO (Figures 6B, C) and KEGG pathways (Figure 6D) was conducted. High-CMLHMS tumors were enriched in pathways associated with muscle contraction, response to copper ions, cell-substrate junctions, and collagen-containing extracellular matrix remodeling, as well as actin binding to the extracellular matrix. These features suggest an extensive remodeling of the tumor microenvironment, facilitating invasion and metastatic dissemination. In contrast, low-CMLHMS tumors showed significant enrichment in cAMP signaling, retinol metabolism, and drug metabolism. These pathways are indicative of a phenotype attuned to stress adaptation, detoxification, and metabolic regulation, reflecting reliance on intrinsic cellular homeostasis mechanisms. The molecular divergence between the two subgroups highlights distinct vulnerabilities. High-CMLHMS tumors, driven by cytoskeletal reorganization, extracellular matrix interactions, and oxidative metabolism, may be particularly susceptible to therapies targeting actin dynamics or matrix remodeling. Meanwhile, low-CMLHMS tumors, with their reliance on hormonal and metabolic pathways, present opportunities for interventions modulating androgen signaling, metabolic flux, or stress response pathways.

Taken together, these findings illuminate two biologically distinct subtypes of PCa stratified by CMLHMS, with high-CMLHMS tumors exhibiting a more invasive and proliferative phenotype, while low-CMLHMS tumors are characterized by metabolic and hormonal adaptability. This molecular stratification offers a framework for tailored therapeutic strategies targeting subtype-specific vulnerabilities.

### CMLHMS score correlates with PCa progression to CRPC

**FIGURE 7:**
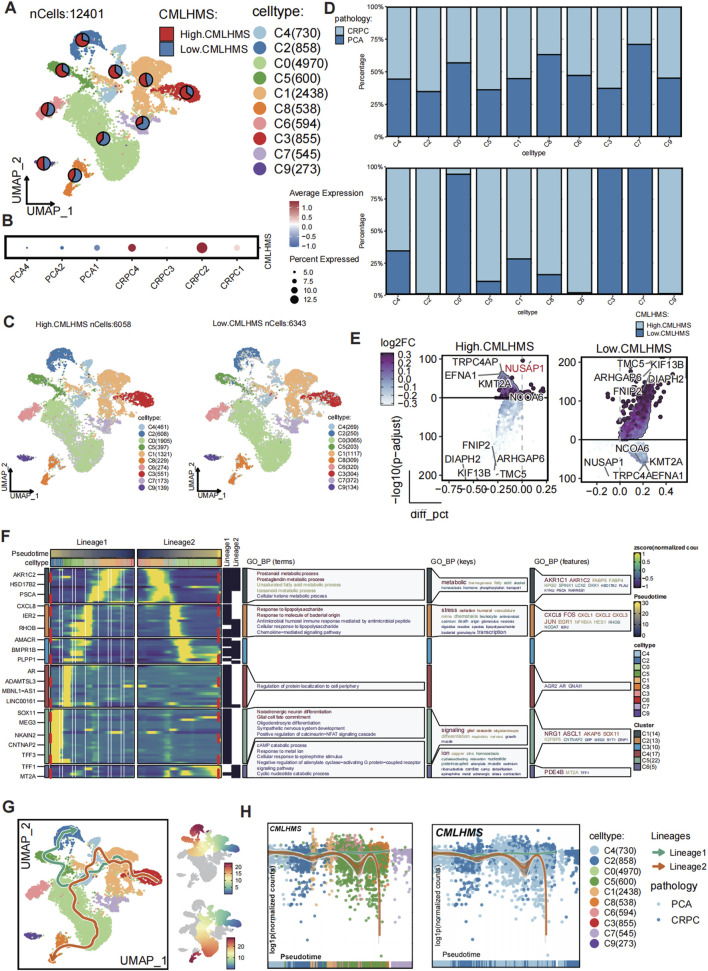
Single-cell analysis of CMLHMS and slingshot pseudotime analysis in PCa. **(A)** UMAP visualization of 12,401 epithelial cells from primary PCa and CRPC samples, divided into ten subclusters. **(B)** Dotplot showed the CMLHMS scores in CRPC tissues and primary PCa. **(C)** Cells are stratified into high- and low-CMLHMS groups based on the mean CMLHMS score. **(D)** Proportion analysis. **(E)** Differential expression analysis identifies key marker genes for high-CMLHMS and low-CMLHMS epithelial cells. **(F, G)** Slingshot trajectory analysis reconstructs two major differentiation lineages, Lineage 1 (metabolism-associated) and Lineage 2 (stress-responsive), highlighting their distinct biological roles. **(H)** Lineage analysis shows the association between CMLHMS scores Lineage 1 and Lineage 2.

To validate the role of the CMLHMS in PCa progression, we performed single-cell RNA sequencing analysis on 12,401 epithelial cells derived from seven patients, including three with primary PCa and four with castration-resistant prostate cancer (CRPC). Clustering analysis divided these epithelial cells into ten distinct subclusters (Figure 7A; Supplementary Figures S3A, B), and CMLHMS scores were calculated for each cell.

CRPC tissues exhibited significantly higher CMLHMS scores compared to primary PCa tissues, illustrating a strong correlation between elevated CMLHMS scores and the development of CRPC (Figure 7B). Cells were further stratified into high- and low-CMLHMS groups based on mean CMLHMS scores (Figure 7C; Supplementary Figure S3C). Proportional analysis revealed that subclusters C3, C1, C2, C4, C5, C6, and C9 were enriched in CRPC components, while subclusters C1, C2, C4, C5, C6, C8, and C9 exhibited elevated CMLHMS scores, consistent with the observed association between CMLHMS scores and CRPC progression (Figure 7D; Supplementary Figure S3D). Differential gene expression analysis identified unique molecular signatures for high- and low-CMLHMS epithelial cells. The high-CMLHMS group exhibited upregulation of genes such as TRPC4AP, NUSAP1, EFNA1, KMT2A, and NCOA6, which are implicated in chromatin remodeling, cell proliferation, and tumor progression. Conversely, the low-CMLHMS group demonstrated elevated expression of genes including TMC5, KIF13B, ARHGAP6, DIAPH2, and FNIP2, which are involved in cytoskeletal organization, intracellular transport, and stress response (Figure 7E).

To further dissect the developmental dynamics of epithelial cells, a pseudotime trajectory analysis was performed using Slingshot (Figures 7F, G). Two major differentiation lineages (Lineage 1 and Lineage 2) were reconstructed, originating from a shared progenitor state and diverging into distinct cellular states along pseudotime. Lineage 1 was characterized by the upregulation of genes involved in metabolic processes, including AKR1C2, HSD17B2, and FABP5, associated with prostaglandin metabolism, unsaturated fatty acid metabolism, and cellular keto metabolism. This lineage reflects a metabolic shift towards energy production and cell growth, consistent with the high-CMLHMS phenotype. Along this trajectory, the proportion of high-CMLHMS cells gradually increased, indicating a direct link between elevated metabolic activity and aggressive tumor progression. Lineage 2, on the other hand, showed a stress-responsive phenotype, with enrichment of immune signaling pathways and cellular responses to external stimuli. This lineage was marked by genes such as IFIH1, STAT1, and IRF7, highlighting adaptation to inflammatory or hostile microenvironments. Along this pathway, the proportion of low-CMLHMS cells increased, suggesting that these cells rely on stress response mechanisms rather than metabolic reprogramming.

Together, these findings reveal a clear dichotomy in epithelial cell differentiation trajectories, with high-CMLHMS cells associated with a metabolism-driven phenotype (Lineage 1) and low-CMLHMS cells exhibiting a stress-adaptive phenotype (Lineage 2) (Figure 7H). This heterogeneity highlights distinct molecular programs underlying PCa progression, particularly the transition to CRPC, and provides insights into potential therapeutic targets tailored to these phenotypes.

Collectively, the correlation between high CMLHMS scores and CRPC progression underscores the metabolic plasticity of high-CMLHMS cells as a key driver of aggressive disease. Conversely, the stress-adaptive nature of low-CMLHMS cells suggests distinct vulnerabilities, offering new opportunities for subtype-specific therapeutic interventions.

### Differential drug sensitivities between high- and Low-CMLHMS PCa groups

**FIGURE 8:**
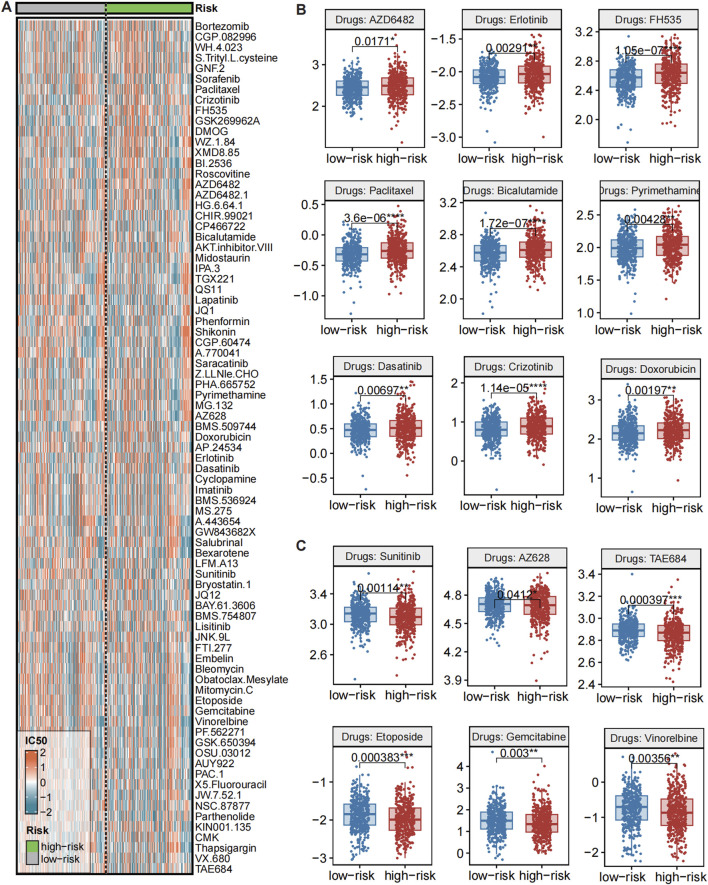
Evaluations of drug sensitivity between high and low CMLHMS groups. **(A)** Heatmap showed the IC50 of drugs from GDSC across CMLHMS groups. **(B)** Comparison of IC50 between high and low CMLHMS groups (low CMLHMS sensitive). **(C)** Comparison of IC50 between high and low CMLHMS groups (high CMLHMS sensitive).

Emerging evidence has unveiled the molecular heterogeneity between high- and low-CMLHMS PCa subtypes, suggesting that these differences may influence therapeutic responses. To investigate this, we evaluated drug sensitivity profiles between the two groups using the Genomics of Drug Sensitivity in Cancer (GDSC) database (Figure 8A). Drug responsiveness was assessed by comparing IC50 values, where lower IC50 values indicate higher drug sensitivity.

Several drugs exhibited significant differential responses, highlighting potential therapeutic implications. As showed in Figure 8B, high-CMLHMS tumors demonstrated enhanced sensitivity to several targeted therapies, as reflected by significantly lower IC50 values for: AZD6482 (PI3K Inhibitor, *P* = 0.0171), Erlotinib (EGFR Inhibitor, *P* = 0.00291), FH535 (β-catenin/PPAR Inhibitor, *P* = 1.05e-07), Dasatinib (BCR-ABL/Src Kinase Inhibitor, *P* = 0.00697), TAE684 (ALK Inhibitor, *P* = 0.000397), AZ628 (BRAF Inhibitor, *P* = 0.0412), indicating better response to those drugs. These findings suggest that tumors with high CMLHMS scores, driven by proliferative and metabolic pathways as revealed in prior pathway enrichment analyses, may be particularly susceptible to therapies targeting growth factor signaling, kinase activity, and metabolic regulators.

In contrast, low-CMLHMS tumors exhibited greater sensitivity to agents targeting cytoskeletal integrity, androgen signaling, and stress-response mechanisms. Drugs with significantly lower IC50 values in the low-CMLHMS group included: Paclitaxel (Microtubule Inhibitor, *P* = 3.6e-06), Bicalutamide (Androgen Receptor Antagonist, *P* = 1.72e-07), Pyrimethamine (Antifolate, *P* = 0.00428), Sunitinib (VEGFR/PDGFR Inhibitor, *P* = 0.00114), Etoposide (Topoisomerase Inhibitor, *P* = 0.00383), Gemcitabine (Nucleoside Analog, *P* = 0.003), Vinorelbine (Microtubule Inhibitor, *P* = 0.00356) (Figure 8C). These results align with the stress-adaptive, immune-regulated phenotype of low-CMLHMS tumors, highlighting their reliance on cytoskeletal dynamics, DNA damage repair, and hormonal pathways.

The observed drug sensitivities reflect the underlying molecular characteristics and pathway dependencies of the two subgroups. High-CMLHMS tumors, characterized by activation of proliferative and metabolic pathways, exhibited pronounced sensitivity to PI3K, EGFR, and β-catenin/PPAR inhibitors, which directly target key drivers of their aggressive phenotype. Conversely, low-CMLHMS tumors, which rely on adaptive stress responses and immune modulation, responded more favorably to microtubule inhibitors, androgen receptor antagonists, and topoisomerase inhibitors, consistent with their distinct cellular vulnerabilities.

These findings emphasize the potential for personalized treatment strategies in PCa. While high-CMLHMS tumors may benefit from targeted therapies focused on growth factor signaling and metabolic regulation, low-CMLHMS tumors appear more responsive to agents targeting hormonal signaling, cytoskeletal dynamics, and DNA replication stress. This stratification provides a framework for optimizing therapeutic regimens based on CMLHMS scores, advancing precision oncology in prostate cancer.

## Discussions

PCa is a highly heterogeneous disease with significant variability in molecular, cellular, and clinical behavior. The therapeutic drug resistance heterogeneity of prostate cancer is multifaceted, encompassing metabolic alterations and interactions with the immune system. For instance, Zhou et al. (2023) discovered that circular RNA circROBO1 promotes prostate cancer growth and contributes to drug resistance to enzalutamide by accelerating glycolysis. Additionally, Ye et al. (2024) emphasized the intricate relationship between the immune microenvironment and tumor development.

Understanding this heterogeneity is critical for optimizing therapeutic strategies and improving patient outcomes (Gillessen et al., 2025; Han et al., 2024; Pan et al., 2024). In this study, we leveraged integrative multi-omics analysis and machine learning to develop the CMLHMS, a novel metric to stratify PCa subtypes based on global histone modification patterns. The combination of Lasso and plsRcox offers a balanced approach that not only selects the most relevant features but also reduces the dimensionality of the data, addressing issues of overfitting and multicollinearity. This integration enhances the prediction accuracy of the model, rendering it particularly suitable for survival analysis in complex tumor datasets, such as those pertaining to prostate cancer. By merging these two methodologies, our model retains a manageable quantity of key features while effectively capturing intricate relationships within the data, thereby facilitating more reliable and robust predictions of prostate cancer prognosis. Our findings revealed profound distinctions in biological processes, treatment sensitivities, and disease progression between high- and low-CMLHMS groups, providing novel insights into the epigenetic landscape of PCa and its clinical implications.

Histone modifications are critical regulators of chromatin structure and gene expression, influencing key cellular processes such as proliferation, differentiation, and apoptosis. Aberrant histone modifications have been implicated in the initiation and progression of various cancers, including PCa (Feinberg et al., 2016; Dawson and Kouzarides, 2012). Our study demonstrates that PCa with high CMLHMS scores is characterized by the activation of proliferative and metabolic pathways, including MYC targets, oxidative phosphorylation, and the G2M checkpoint. These pathways are well-known drivers of aggressive tumor phenotypes and are associated with advanced disease stages (Mishra et al., 2024; Grasso et al., 2012). Conversely, low CMLHMS tumors showed enrichment in stress-adaptive and immune-regulatory pathways, such as androgen and estrogen responses and KRAS signaling. These findings align with previous studies indicating that epigenetic regulation plays a pivotal role in determining tumor aggressiveness and therapeutic resistance (Sinha et al., 2019; Yang et al., 2022).

The observed differences in pathway activation underscore the impact of histone modifications on PCa heterogeneity. High-CMLHMS tumors may represent a phenotype driven by deregulated chromatin states that promote rapid cell cycling and metabolic reprogramming, whereas low-CMLHMS tumors appear to rely on epigenetic mechanisms that facilitate stress adaptation and immune evasion. These distinct molecular profiles highlight the potential of histone modification patterns as biomarkers for PCa subtype classification and prognosis.

Castration-resistant prostate cancer (CRPC) represents a lethal stage of PCa that arises despite androgen deprivation therapy (ADT). The transition to CRPC involves complex molecular changes, including epigenetic reprogramming (Mandigo et al., 2022; Sharma et al., 2010). Using single-cell RNA sequencing, we showed that CRPC tissues exhibited significantly higher CMLHMS scores compared to primary PCa tissues, suggesting that elevated histone modification activity is a hallmark of CRPC progression (Wang et al., 2020). Trajectory analysis further revealed two distinct differentiation lineages among epithelial cells: Lineage 1, associated with high CMLHMS scores, exhibited upregulation of metabolic genes and pathways related to prostaglandin metabolism and unsaturated fatty acid metabolism, reflecting a shift towards energy production and tumor growth. In contrast, Lineage 2, enriched in low CMLHMS scores, demonstrated a stress-responsive phenotype characterized by immune signaling and adaptation to hostile microenvironments.

These findings provide a mechanistic link between histone modifications and CRPC development. The metabolic phenotype of high-CMLHMS cells may confer a selective advantage under androgen-deprived conditions, promoting tumor progression and therapeutic resistance. On the other hand, the stress-adaptive phenotype of low-CMLHMS cells highlights the role of immune evasion and microenvironmental interactions in CRPC biology. This dichotomy underscores the importance of epigenetic plasticity in driving PCa heterogeneity and resistance to standard therapies (Pan et al., 2024; Ni et al., 2024).

Our analysis revealed significant differences in drug sensitivities between high- and low-CMLHMS tumors, reflecting their distinct molecular characteristics. High-CMLHMS tumors exhibited greater sensitivity to targeted therapies, including PI3K inhibitors (AZD6482), EGFR inhibitors (Erlotinib), β-catenin/PPAR inhibitors (FH535), and kinase inhibitors such as Dasatinib and TAE684. Obviously, the CMLHMS score is closely associated with the biological characteristics of the tumor, which subsequently influences its response to various pharmacological treatments. Tumors with a high CMLHMS score typically demonstrate enhanced activation of cell proliferation and metabolic pathways (Nadiminty et al., 2013), including MYC targets, oxidative phosphorylation, and angiogenesis (Liu et al., 2014), rendering them more susceptible to therapies that target these proliferation and metabolic pathways (Anwaier et al., 2022; Zhu et al., 2022). Conversely, low CMLHMS tumors primarily depend on stress adaptation and metabolic regulation, and they exhibit a more favorable response to drugs that target cytoskeletal dynamics, DNA repair, and androgen signaling. Targeting these pathways could provide a therapeutic advantage in high-CMLHMS patients.

Conversely, low-CMLHMS tumors demonstrated enhanced sensitivity to drugs targeting cytoskeletal dynamics (e.g., Paclitaxel, Vinorelbine), androgen signaling (e.g., Bicalutamide), and DNA damage repair (e.g., Etoposide, Gemcitabine). The stress-adaptive and immune-regulatory phenotype of low-CMLHMS tumors may render them more vulnerable to therapies that disrupt cellular homeostasis or exploit DNA replication stress. These results are consistent with previous studies highlighting the therapeutic potential of microtubule inhibitors and androgen receptor antagonists in less aggressive PCa subtypes (Bian et al., 2024; Ni et al., 2023). The differential drug sensitivities observed in this study provide a rationale for tailoring treatment strategies based on CMLHMS scores. High-CMLHMS patients may benefit from targeted therapies that disrupt proliferative and metabolic pathways, while low-CMLHMS patients may respond better to cytoskeletal inhibitors and hormone-based treatments. This stratified approach represents a step forward in the development of precision medicine for PCa.

One of the key strengths of this study is the integration of multi-omics data and machine learning to develop the CMLHMS model. This approach enabled us to capture the complexity of histone modification patterns and their functional implications in PCa. Additionally, the use of single-cell RNA sequencing provided a high-resolution view of tumor heterogeneity and allowed us to identify distinct differentiation trajectories associated with CMLHMS scores. Collectively, the innovation of this article lies in its focus on holistic histone modifications, utilizing multiple batch cohorts to evaluate comprehensive patterns. This approach offers a novel perspective for prostate cancer research, distinguishing it from previous literature on the subject (Zhu et al., 2025).

However, several limitations should be acknowledged. First, the sample size for single-cell analysis was relatively small, particularly for CRPC tissues, which may limit the generalizability of our findings. Second, while the GDSC database provided valuable insights into drug sensitivities, experimental validation of these predictions in preclinical or clinical settings is necessary. Third, the functional roles of key genes identified in high- and low-CMLHMS groups (e.g., TRPC4AP, NUSAP1, ARHGAP6) remain to be elucidated. Future studies should focus on validating these findings and exploring the underlying mechanisms.

This study lays the groundwork for several future research avenues. First, the CMLHMS model should be validated in larger, independent cohorts, including prospective clinical trials, to confirm its prognostic and predictive value. Second, mechanistic studies are needed to investigate the functional roles of histone modifications and key genes in driving PCa heterogeneity and CRPC progression. Third, preclinical studies should evaluate the efficacy of targeted therapies identified in this study, such as PI3K inhibitors for high-CMLHMS tumors and microtubule inhibitors for low-CMLHMS tumors. Finally, exploring combination therapies that target both proliferative and stress-adaptive phenotypes may help overcome therapeutic resistance and improve patient outcomes.

## Conclusion

In conclusion, this study highlights the critical role of histone modifications in shaping PCa heterogeneity and progression. By integrating multi-omics data and machine learning, we developed the CMLHMS model, which provides a novel framework for understanding the epigenetic landscape of PCa. The findings reveal distinct molecular subtypes with unique biological processes, therapeutic sensitivities, and clinical implications. These insights pave the way for precision oncology strategies that tailor treatments based on histone modification patterns, offering new hope for improving outcomes in PCa patients.

## Data Availability

The original contributions presented in the study are included in the article/Supplementary Material, further inquiries can be directed to the corresponding authors.

## Supplementary material

SUPPLEMENTARY FIGURE S1 Removing batch effects.

SUPPLEMENTARY FIGURE S2 Survival analysis for CDR2L, NUSAP1, and ZNF467 among TCGA-PRAD **(A)**, GSE70770 **(B)**, and MSKCC **(C)** cohorts. **(D)** Differentiallly expressed genes between high and low CMLHMS groups, which was performed using limma algorithms, log2fc > 1 and padj < 0.05 was considered as upregulation.

SUPPLEMENTARY FIGURE S3 Single cell analysis. **(A)** UMAP showed the distribution of patients before harmony. **(B)** UMAP showed the distribution of patients after harmony. **(B)** Distribution of samples among high and low CMLHMS epithelial. **(C)** Distribution of groups among high and low CMLHMS epithelial. **(D)** The Sankey plot illustrated the distribution of epithelial subtypes between high and low CMLHMS epithelial.
