# Supplementary figures and images for: Integrative multi-omics analysis and machine learning refine global histone modification features in prostate cancer
Source: Front Mol Biosci. 2025 Mar 12;12:1557843. doi: 10.3389/fmolb.2025.1557843 (PMC11936803; doi:10.3389/fmolb.2025.1557843)

A

Risk genes in TCGA-PRAD

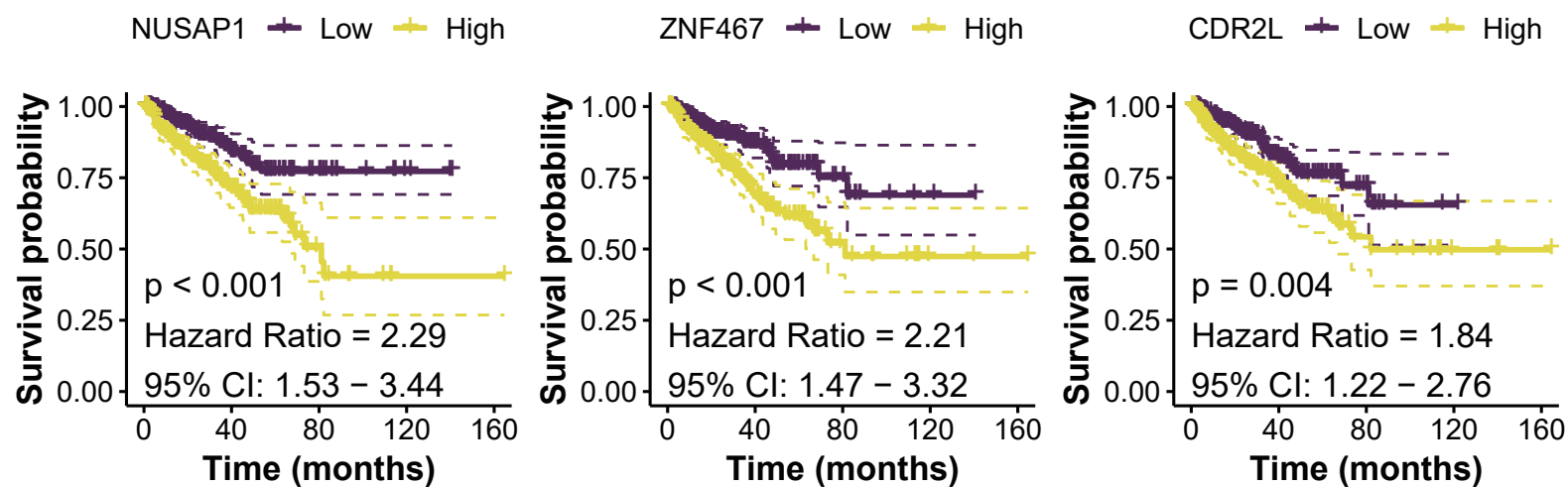

B

Risk genes in GSE70770

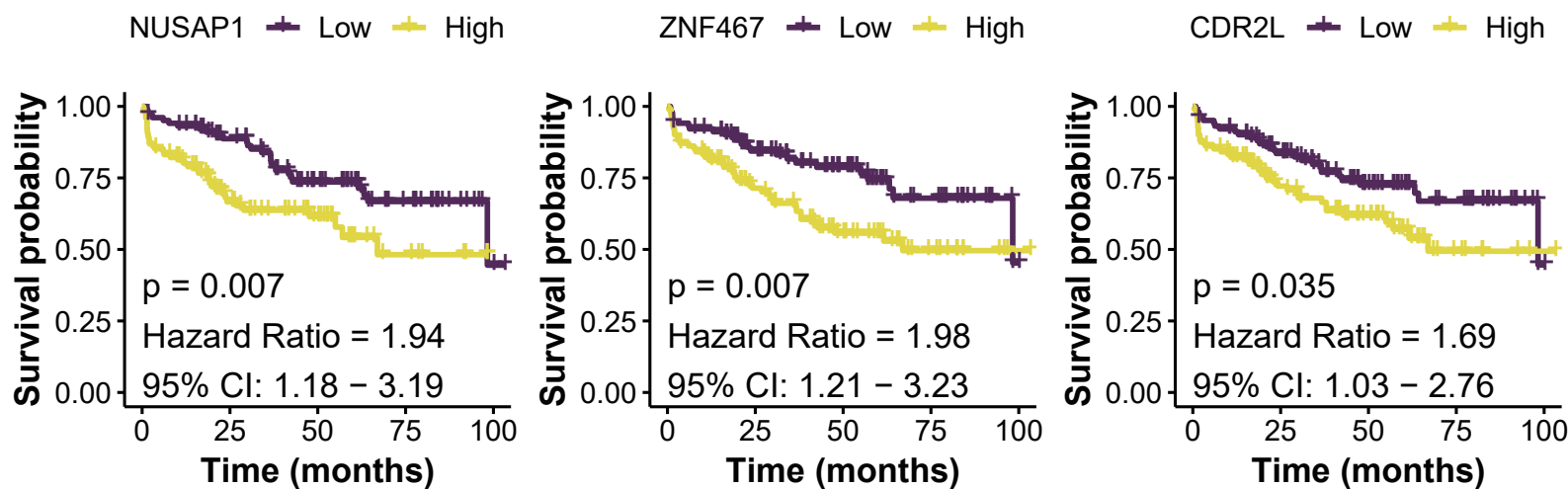

C

Risk genes in MSKCC

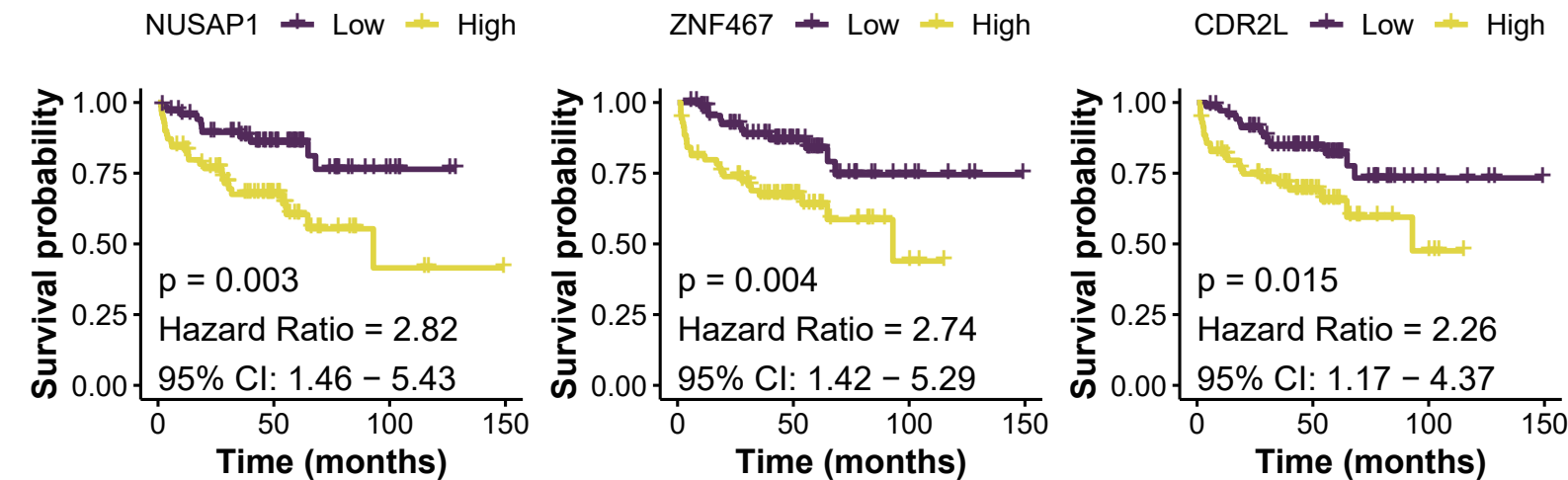

D

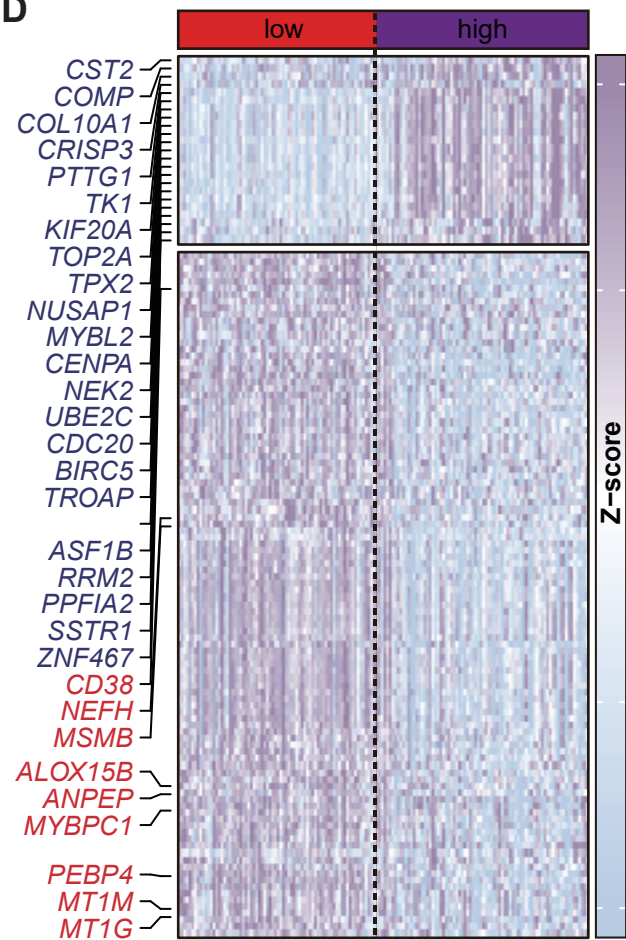

Supplement: Supplementary file 1 [file Image2.pdf]

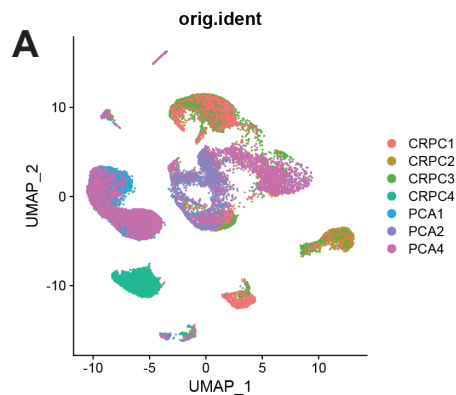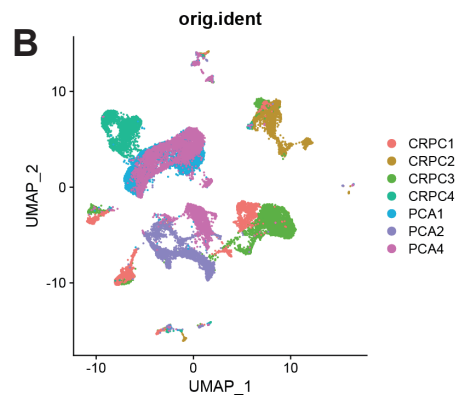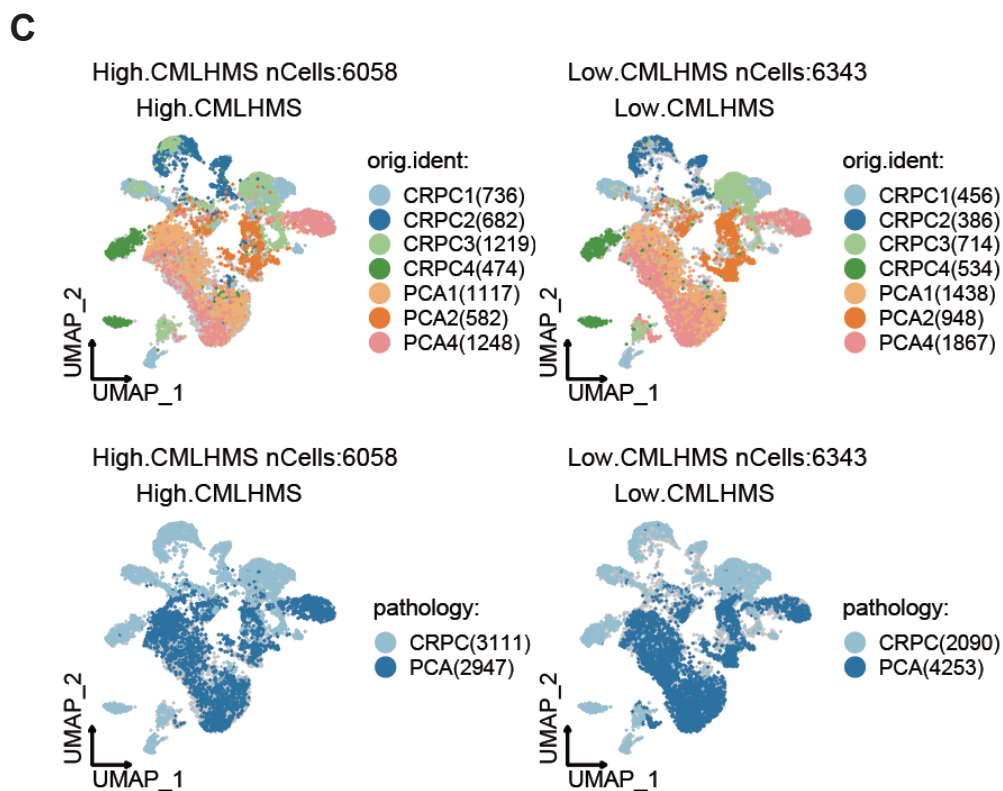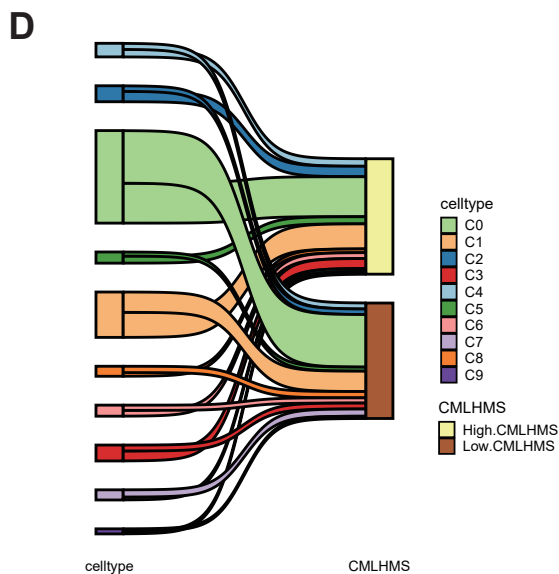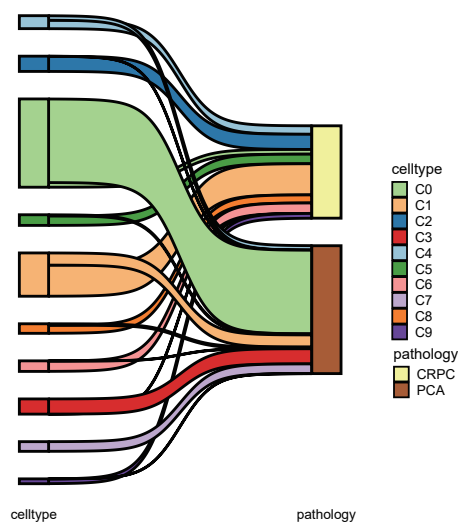

Supplement: Supplementary file 2 [file Image3.pdf]

Raw PCA for combined expression profile

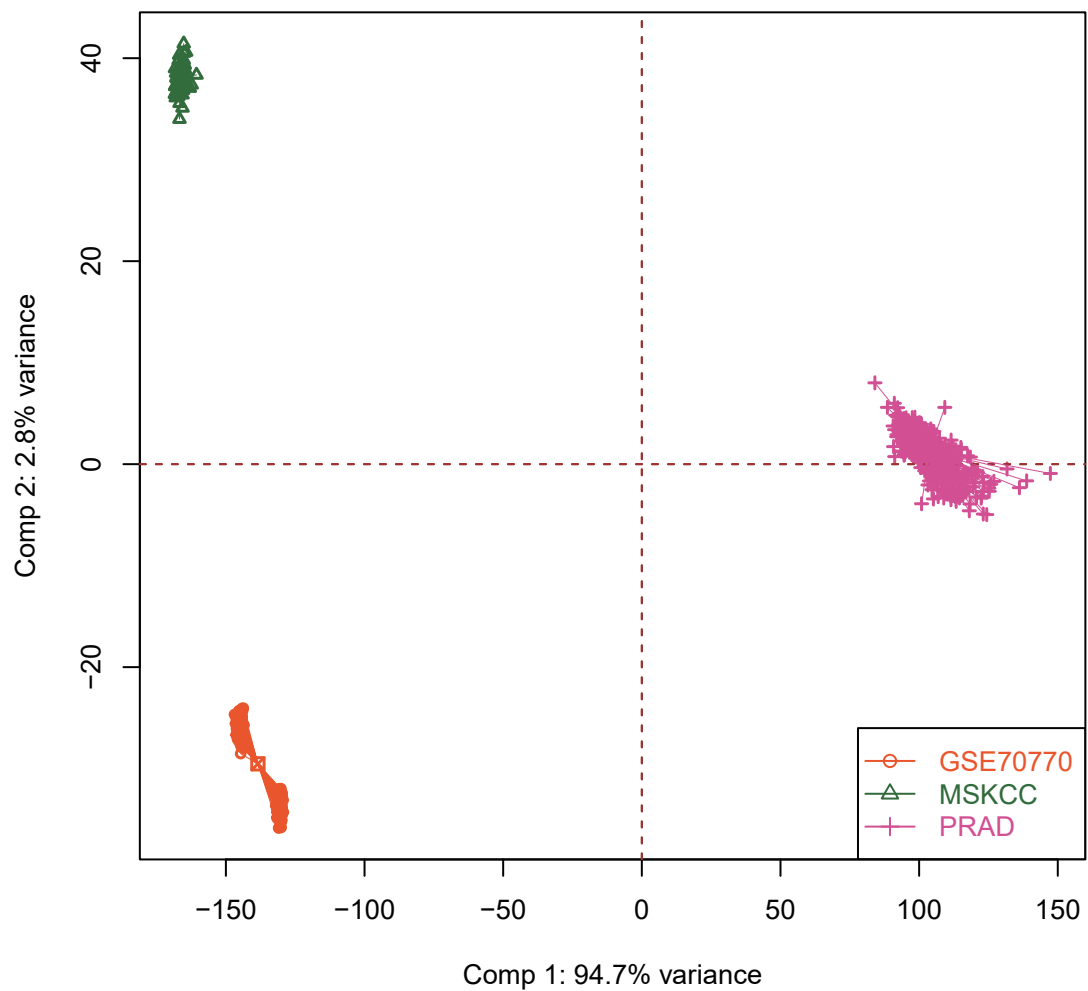

Combat PCA for combined expression profile

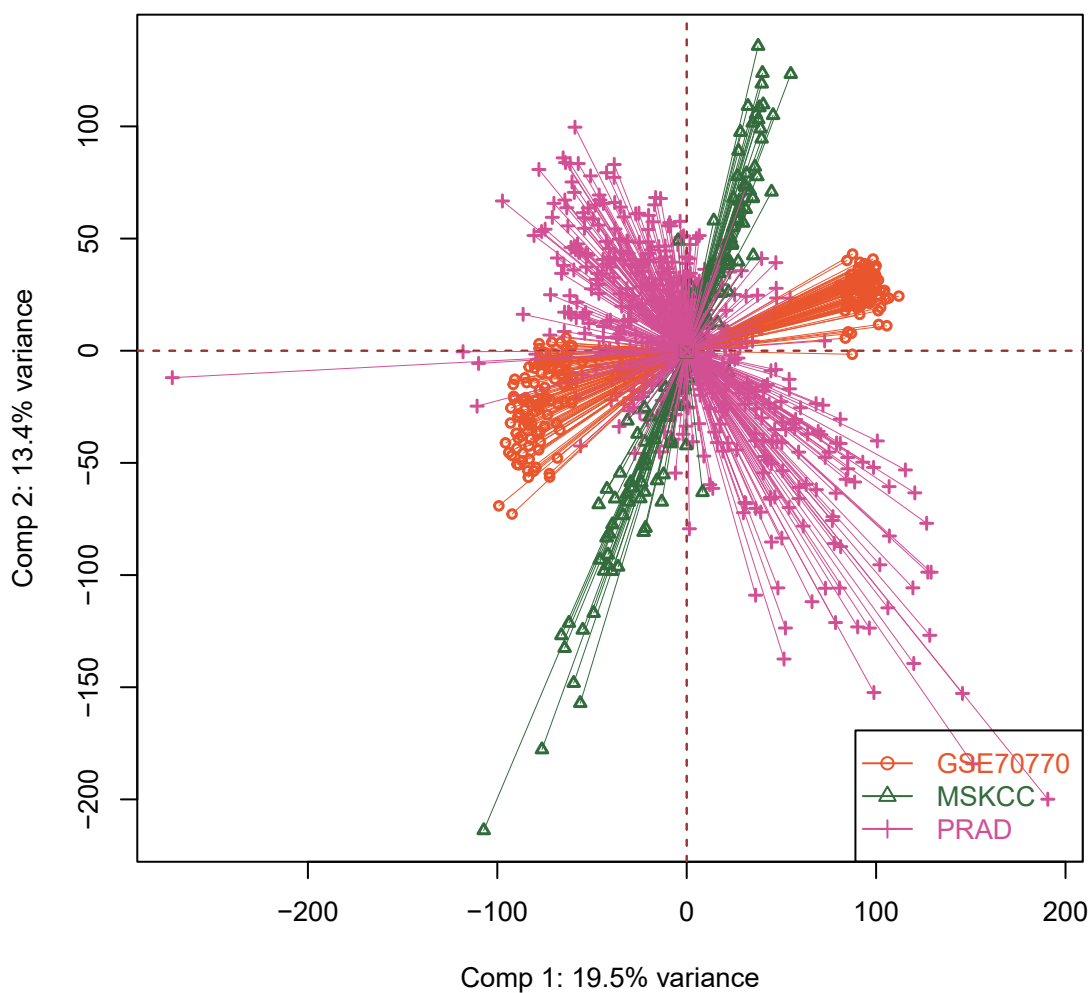

Supplement: Supplementary file 3 [file Image1.pdf]
